# Working with patients to optimise cataract outcomes

**Published:** 2016

**Authors:** Nick Astbury, Ebby Adekhera, Lily A Nyamai

**Affiliations:** Clinical Senior Lecturer: International Centre for Eye Health, London School of Hygiene and Tropical Medicine, London, UK.; Nursing Officer: Sabatia Eye Hospital, Wodanga, Kenya.; Tutorial Fellow: Department of Ophthalmology, University of Nairobi, Nairobi, Kenya. **lilynyamai@gmail.com**

**Figure:**
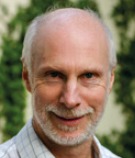
Nick Astbury

**Figure:**
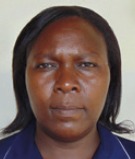
Ebby Adekhera

**Figure:**
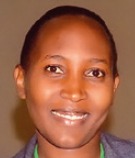
Lily A Nyamai

One of the delights of ophthalmology is to witness the joy on the face of a patient with cataract when the dressing is taken off and sight has been restored. Unfortunately, for some patients, the result does not live up to their expectations. Despite cataract surgery being one of the most successful surgical interventions available, there is evidence that the visual outcome of cataract surgery in sub-Saharan Africa is not always good (defined as a VA of 6/18 or better). The proportion of good outcomes range from only 23% up to 70%, failing to reach the WHO target of 85% or better.^1^

A good outcome is crucial for the individual patient, but will also have a wider impact on the community. In sub-Saharan Africa, for example, uneasiness about surgery can mean that patients stay away – more so if they hear about an operation that was not successful. Good outcomes in cataract surgery, in those brave enough to undergo the procedure, are therefore essential to encourage other people with poor vision from the community to come forward for examination and treatment.

In order to optimise good outcomes, patients need to have relevant information. They must have confidence in the eye service and in the people providing it, so that they will be willing to attend follow-up visits and to come back immediately if they notice anything wrong after the operation.

It is helpful to have a team member who speaks the language of patients as this can help to increase people's understanding of any information being shared and boost their trust in the eye service.

## Before surgery

Patients and their families must be given advice and counselling about the operation, including what happens before, during and after. They should then sign an informed consent form. It is our responsibility to ensure that the patient understands – in straightforward terms – what is going to happen and what this means for them and their eye health. We must also take time to address any fears, doubts and myths about cataract surgery.

**Figure:**
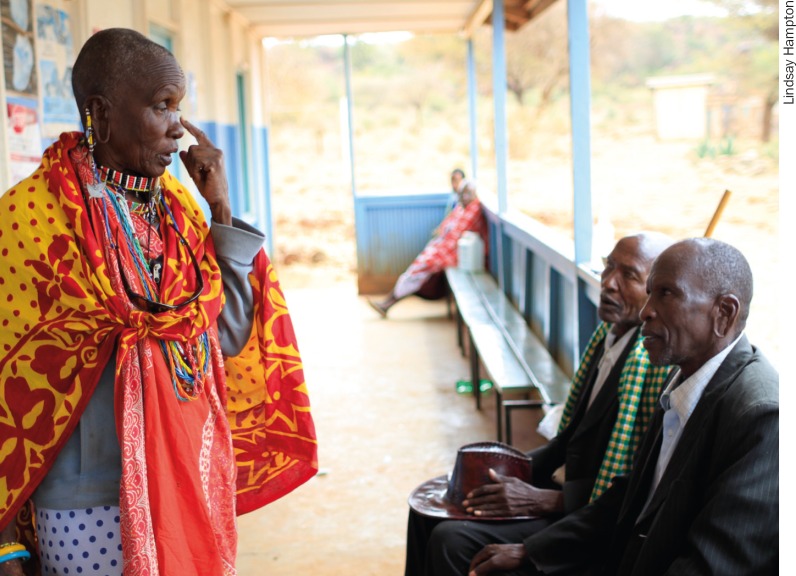
A woman tells new patients about her cataract operation. KENYA

It is important to ensure that patients and their families have realistic expectations about their vision after surgery. They must understand the risks and benefits, including the potential effect of different complications on their eyesight. A disappointed patient is not a good advertisement for our service.

Our patients also have a role to play in alerting the eye team to potential complications at an early stage. Before surgery, explain to patients how their eyes will look and feel after a successful operation, including what level of discomfort is normal at the different stages of recovery. Encourage them to speak with an eye team member if they experience anything that concerns them.

## After surgery

After the operation, patients should be given clear instructions about how to look after their operated eye when they are back at home (see panel on page 24). Give specific information about follow-up visits (where to go, when, and at what time) and ensure patients know how to get in touch with the eye clinic or their surgeon if they have any signs or symptoms that can indicate a complication (worsening sight, increasing pain, redness, swelling or discharge).

Discussion groups for patients, based on their gender and initial visual outcome, offer an opportunity to talk about coping with the challenges of self-care and follow-up appointments, which may be different for each individual. Giving patients an opportunity to attend such groups may help to allay fears and can give them an opportunity to ask questions if they are uncertain about anything.

## Discharge

Before discharging a patient, check that they have all of the following:

- Instruction sheet to take home
- Clinic contact details
- Eye shield (if available)
- Eye drops and instructions for storage and use
- Painkillers to use at home
- A follow-up appointment date and time.

## Follow-up

We recommend that cataract patients are followed up and examined as follows:

- **The day after surgery (day 1),** in the hospital.
- **4–8 weeks after surgery.** This visit is important, as it is also the time to conduct postoperative refraction. Actively encourage patients to attend, for example by including the visit in the price of the cataract operation.

Transport may be a barrier for some patients and it may be helpful to conduct follow-up appointments in primary health care centres in the community.

## Postoperative refraction

Postoperative refraction and provision of spectacles (if needed) are essential to ensure the best possible visual outcome for a patient. This is important because satisfied patients will encourage others in the community to undergo cataract surgery.

During the 4–8-week follow-up appointment, refract both eyes and accurately check the visual acuity. Make sure you understand the patient's refractive needs (e.g. their ideal working distance).

If there is no intraocular lens, carefully check the back vertex distance and centring of the spectacles.

**Instructions for patients**

Even though you may feel well after surgery, you have had a big operation. You should take care of yourself and allow your eye to heal properly.

**Dos**

- Clean eyelids morning and evening with a moist, clean face cloth, avoiding pressure on the eyeball.
- If possible, protect the operated eye for the first week by wearing an eye shield when sleeping and sunglasses or prescription spectacles during the day.
- Instil eye drops as prescribed.
- Follow a normal diet after surgery with enough water and fibre/roughage (from fruit, vegetables and whole grains) to avoid constipation.
- Resume your regular medications, including any prescribed eye drops, immediately.
- **Contact the eye clinic in case of worsening sight, increasing pain, redness, swelling or discharge.**
- Keep your follow-up appointments without fail.
- You can wash your hair a day after surgery but avoid soap, water or shampoo entering the eye.
- You may resume sexual activity once you feel comfortable.

**Don'ts**

- Don't wear eye makeup for at least a week, and don't use shop-bought cotton wool balls on your eyelids. These may leave behind particles of cotton, which may attract germs, leading to infection.
- Avoid sleeping on the operated side.
- Do not lift heavy weights above 5 kg for 2 weeks.
- Avoid swimming for 2 weeks.

## References

[1] Blindness and visual impairment due to age-related cataract in sub-Saharan Africa: a systematic review of recent population-based studies. Bastawrous Andrew Dean William H Sherwin Justin C Br J Ophthalmol 2013 97:1237–1243. Originally published online in May 21, 2013. 23696652 10.1136/bjophthalmol-2013-303135

